# Impact of Synchronous Online Physical Education Classes Using Tabata Training on Adolescents during COVID-19: A Randomized Controlled Study

**DOI:** 10.3390/ijerph181910305

**Published:** 2021-09-30

**Authors:** Kwang-Jin Lee, Byungjoo Noh, Keun-Ok An

**Affiliations:** 1 Department of Physical Education, Chungbuk National University, Cheongju 28644, Korea; rhkdwls@cbnu.ac.kr; 2 Department of Kinesiology, Jeju National University, Jeju 63243, Korea; 3 Sports Medicine Major, Division of Sports, Korea National University of Transportation, Chungju 27469, Korea

**Keywords:** adolescents, COVID-19, synchronous online physical education, Tabata training

## Abstract

This study aimed to investigate the effects of online physical education classes, using Tabata training, on middle school students’ physical fitness. Fifty-four adolescents were randomly assigned to either the asynchronous online class group (AOCG, *n* = 24, age: 15.8 ± 0.4 years) or the synchronous online class group (SOCG, *n* = 24 age: 15.9 ± 0.3 years). The online physical education class lasted two days per week for 10 weeks. Recorded video lectures were conducted for the AOCG, and Tabata training for the SOCG, as real-time lecture methods. Baseline and post-online physical education class measures included muscular strength, muscular endurance, flexibility, balance, and cardiorespiratory fitness tests. The results showed that the synchronous online physical education class had a positive effect on the improvement of muscle mass, ankle strength (dorsiflexion), hip strength (abduction, flexion, extension, and external rotation), knee strength (extension and flexion), and balance (Y-balance test) in adolescents. These findings suggest that the physical fitness of adolescents can be sufficiently improved through appropriate online physical education class methods. Further research should focus on developing and evaluating different types of exercises for synchronous online physical education classes as a precautionary measure for the second wave of COVID-19.

## 1. Introduction

The World Health Organization (WHO) declared the global pandemic of Coronavirus Disease-2019 (COVID-19) on 11 March 2020 [1]. The COVID-19 pandemic has resulted in an unusual life, symbolized by the isolation and confinement of wearing a mask and social distancing [2]. To respond to the severity of COVID-19, the Korean government has raised the infectious disease crisis level to “severe”. It has strengthened the early detection of infected patients, screening clinics, and quarantine regulations to block inflow and spread [3]. The government has established and implemented a policy to curb the spread of COVID-19 by applying intensive social distancing rules in religious facilities, PC rooms, and indoor sports facilities to prevent group infection [3]. The Korean Ministry of Education is currently delaying the opening of all kindergarten, elementary, middle, and high schools as well as universities across the country to protect students from COVID-19 [4]. These lockdowns are affecting people’s work, education, travel, and leisure, thus including their level of physical activity (PA) and sedentary behavior (SB) [5]. Reduced the body composition, muscular strength, and balance ability of adolescents [6], increased the obesity rate [7], and affected mental health [8] was reported severe concerns during non-face-to-face classes due to COVID-19.

Lifestyle behaviors, such as PA and SB among adolescents, may be affected by school closures and long-term confinement at home during the COVID-19 pandemic [9]. Likewise, in Korea, isolation from the family for a long time and the delay in normal school life pose a great threat to the physical and mental health and daily life of middle-school adolescents [10]. In general, schools are well equipped to promote health-related physical fitness and PA among adolescents, as they spend 6–7 h a day at school for 40 weeks of the year; there are facilities, equipment, curricula, and physical education (PE) experts who can intervene in health promotion [11]. Furthermore, these environments can help adolescents meet the recommended daily 60 min of moderate-vigorous physical activity (MVPA) and develop the knowledge, skills, and confidence to continue PA in the future [12]. MVPA is important for maintaining and improving health-related fitness (i.e., body composition, cardiorespiratory fitness, muscular strength, endurance, and flexibility) [13]. Therefore, the change in PE class methods, i.e., an online method of class delivery in response to school closure, may lead to a decline in the physical fitness of adolescents.

Meanwhile, the school lockout policy has forced adolescents worldwide to participate in physical education classes online instead of on the playground [14]. The change is making physical education teachers adapt quickly to the new class environment, which demands a devoted effort [15]. In addition, the support of the government and educational institutions through policies is required, due to the deterioration in adolescent health [9,16]. In France, teachers have strengthened all policy capabilities to increase students’ out-of-school PAs; however, in Italy and Turkey, guidelines have proposed a reduction in out-of-school PA [17]. The Korean Ministry of Education has provided physical education teachers with options for online physical education classes to choose physical activity types: (1) asynchronous online class (content-focused class), (2) synchronous online class (real-time bilateral class), and (3) assignment-oriented class [4]. However, teachers in charge of practical classes, such as PE, have difficulty in selecting an online class type that can cope with field-oriented active classes. Additionally, the selection of teaching methods in PE depends on the PE teachers; therefore, the influence on students may differ according to the nature of the online PE classes. Therefore, based on the online teaching method chosen, PA will be emphasized or restricted.

In various online physical education methods, considerations for improving the physical fitness of adolescents, especially MVPA activities, include limited class time (40 min; including warming up, the main exercise, and cooling down), securing an exercise space (possible to perform in a small space), teacher expertise, and real-time feedback [15,18]. Tabata exercise is considered a suitable form of exercise for adolescents who need MVPA activity because it can be performed at high intensity in a short period while including the four conditions mentioned above [19]. In previous studies, it was reported that face-to-face Tabata exercise [20] and online home-based exercise programs [21] have a positive effect on increasing physical activity in adolescents. PE is the only subject that includes PA in the Korean middle school education curriculum [22,23]. PA during PE classes can significantly influence the growth of adolescents, such as improving their physical strength, health, and athletic ability. Thus, it is necessary to investigate the effects of online PE classes during COVID-19. Therefore, this study aimed to investigate the effects of an online PE class on middle-school students’ physical fitness.

## 2. Materials and Methods

### 2.1. Participants

Sixty middle school students (power > 80% and error = 0.05) volunteered to participate (30 males, 30 females), with 24 of them randomly assigned to the asynchronous online class group (AOCG, age: 15.8 ± 0.4 years, weight: 61.8 ± 9.5 kg, and height: 165.4 ± 8.1 cm) and 24 were randomly assigned to the synchronous online class group (SOCG, age: 15.9 ± 0.3 years, weight: 60.7 ± 8.3 kg, and height: 165.9 ± 6.7 cm), according to a computer-generated randomization sequence (see Figure S1). The inclusion criteria for the study were as follows: (a) participants were not attending school, and (b) subjects who had no experience with school and private PE classes were recruited during the COVID-19 pandemic. Before the test, participants with body temperatures higher than 37 °C were excluded to prevent the risk of COVID-19. Twelve participants dropped out of the study (Figure S1). The selected participants had not been diagnosed with any orthopedic injury within the last three months. All participants and their parents signed an informed consent form that was approved by the Institutional Review Board (CBNU-202012-HR-0196). This study was registered with the Clinical Research Information Service (Korea, https://cris.nih.go.kr; registration number: KCT0006559; accessed date: 9 September 2021). This study followed the Consolidated Standards of Reporting Trials (CONSORT) reporting guidelines.

### 2.2. Measurements

The measurements included body composition and physical fitness, which were used to measure the primary and secondary outcomes. Both outcome measures were conducted at each time point as a baseline and at the 10-week follow-up (immediately after the 10-week intervention). To prevent infection and the spread of COVID-19, the body temperature of all participants was taken, and equipment were disinfected before measurement. Consideration was given to the place, such as the participant’s home, or an individual measurement to minimize movement, preference, and measurement time. The responsible researcher wore body temperature measurements, masks, and sanitary gloves before performing the measurement tasks. A designated place outside the school was used to measure 46 out of 48 participants. The measurements of two participants were taken at their homes as requested by the parents. The body composition was measured with a bioelectrical impedance analyzer (InBody 320, Biospace, Seoul, Korea). Physical fitness was measured in terms of muscular strength, muscular endurance, flexibility, balance, and cardiorespiratory fitness.

#### Measurement of Physical Fitness Test

Physical fitness tests were conducted in four domains: strength, flexibility, balance, and cardiorespiratory fitness. The participants completed the physical fitness tests in the following order:

1. Muscular strength (ankle joint: plantar flexion/dorsiflexion and inversion/eversion; knee joint: flexion/extension; hip joint: abduction/adduction, flexion/extension, and internal/external rotation) was measured, using a manual muscle test with a handheld dynamometer (micro FET2TM, Hoggan Health Industries, Salt Lake City, UT, USA). The maximum isometric force generated for 2 s in each part was measured, and the relative muscle strength value (N·m/kg) divided by the subject’s weight was adopted [6].
2. Muscular endurance was measured through sit-ups. The number of sit-ups performed for 30 s was recorded. During the measurement, if the elbow did not touch the knee or the back did not touch the mat, the sit-up was excluded from the total number performed [24].
3. Flexibility was measured using a sit-and-reach device (Takei, T.K.K. 5103, Tokyo, Japan). The measurement unit was recorded as 0.1 cm [24].
4. Balance was measured using a single-leg balance test with closed eyes and the Y-balance test (YBT) in barefoot conditions. Balance was measured after the hands were placed parallel to the shoulders, and the knee of the non-dominant limb was horizontal to the ground [25]. YBT requires balancing with the dominant leg while reaching as far as possible with the non-dominant leg in three separate directions (anterior—AN, posteromedial—PM, and posterolateral—PL directions). The length of the lower extremity was measured from the anterior superior iliac spine to the medial malleolus. The subject was given a chance to re-measure when they failed to return to the initial position or when the foot touched the ground during the measurement [26]. The composite score was calculated using the following formula [26]:
  (1) $$\mathrm{Composite}\;\mathrm{score}=\frac{\left(\mathrm{Anterior}+\mathrm{Posteromedial}+\mathrm{Posterolateral}\;\mathrm{direction}\right)}{3\times \mathrm{Right}\;\mathrm{limb}\;\mathrm{length}}\times 100$$
5. Cardiorespiratory fitness was measured using the Harvard step test. The Harvard Step Test measured the recovery heart rate at 1 min, 2 min, and 3 min after allowing the subject to climb the step and back down for 3 min, with the step box having a height of 45.7 cm, at a speed of 120 bpm. For heart rate measurement, a portable heart rate monitor (Polar Electro, Technogym, Uusimaa, Finland) was used. The measurement was stopped if the subject requested halting due to issues, such as breathing difficulties, at which time the measurement was stopped and recorded, and the heart rate was measured as in the previous method [27]. The physical efficiency index (PEI) was calculated using the heart rate measured during resting [27]. The PEI was calculated using the following formula:
  (2) $$\mathrm{PEI}\;=\frac{\mathrm{Duration}\;\mathrm{of}\;\mathrm{the}\;\mathrm{exercise}\;\left(s\right)}{\left(2\times \left(\mathrm{first}\;\mathrm{phase}+\mathrm{second}\;\mathrm{phase}+\mathrm{third}\;\mathrm{phase}\;\mathrm{heart}\;\mathrm{rates}\right)\right)}\times 100$$
  Each phase was 60–90 s (first phase heart rates), 120–150 s (second phase heart rates), and 180–210 s (third phase heart rates), respectively.

### 2.3. Online Physical Education Classes

#### 2.3.1. Asynchronous Online Class (Content-Focused Class)

The asynchronous online class is an online PE class in which the video lectures created by the teacher are loaded on the online platform (Google class and education broadcasting system online class), and the students watch the uploaded video. The PE class was conducted twice a week for 40 min across 10 weeks, and the content of the study included basic skills and concepts of volleyball (3 weeks), basic skills and concepts of basketball (3 weeks), Tabata training (2 weeks), and school safety education (2 weeks) (see Table S1).

#### 2.3.2. Synchronous Online Class (Real-Time Bilateral Class)

A synchronous online class is a method in which teachers and students participate in PE classes in real time, using an online platform (Zoom). In our study, the students conducted Tabata training under the guidance of PE teachers. The teacher received three training sessions on suitably applying the Tabata exercise method to the class. Tabata training is a type of high-intensity interval training (HIIT) in which participants exercise for 20 s and rest for 10 s. Eight motions are performed per session, and it consists of three sessions of 14 min. For the present study, each session was given a 1 min rest, and a 10 s low-intensity exercise form (walking in place) was given after 20 s with maximum exercise intensity (RPE 15 or more) [20]. Before exercising, the students performed a warm-up exercise (dynamic stretching) for 10 min and a cool-down exercise (static stretching) for 10 min post-exercise. The time for online class net environment check, attendance check, and notice was included (6 min) since the exercise program applied to the synchronous online class was conducted in a regular class format. The PE teacher motivated the students to perform as many exercise movements as possible. For 10 weeks, 40 min of class was conducted twice a week (see Table S1).

### 2.4. Statistical Analysis

The data were processed using SPSS version 23.0 (SPSS Inc., Chicago, IL, USA) to calculate the descriptive statistics (Mean ± SD). The Shapiro–Wilk test was performed to confirm the normal distribution of the data. A two-way repeated measures ANOVA was conducted to analyze the interaction effect between the group and time taken, according to the online teaching method. A paired t-test was used to verify the changes before and after each group. An independent samples t-test was performed to check the significance level between groups before and after the program intervention. The significance level was set at α = 0.05.

## 3. Results

### 3.1. Changes in Body Composition

The results of the 10 weeks of online PE classes on body composition are shown in Table 1. Body mass index (BMI) showed no interaction effect between time and groups (F = 0.240, *p* = 0.626), and there was a significant difference between the time points (F = 4.204, *p* = 0.046), with no significant difference between the groups (F = 0.563, *p* = 0.457). Body fat showed no interaction effect between time and groups (F = 2.360, *p* = 0.131), and there was a significant difference between the time points (F = 4.587, *p* = 0.038), with no significant difference between the groups (F = 0.234, *p* = 0.631). Muscle mass showed an interaction effect between time and groups (F = 5.426, *p* = 0.024), and there was a significant difference between the time points (F = 4.732, *p* = 0.035), with no significant difference between the groups (F = 0.448, *p* = 0.507).

### 3.2. Changes in Muscle Strength and Muscle Endurance

The results of 10 weeks of online PE classes on muscle strength and endurance are shown in Table 2. The ankle dorsiflexion strength showed an interaction effect between time and group (F = 4.284, *p* = 0.044) but no significant difference between the time points (F = 3.795, *p* = 0.058) and between groups (F = 1.206, *p* = 0.278). The ankle plantarflexion, inversion, and eversion strength showed no interaction effect between time and group (F = 2.015, *p* = 0.163; F = 0.492, *p* = 0.486; F = 2.328, *p* = 0.134), and there was no significant difference between the time point (F = 1.192, *p* = 0.281; F = 0.199, *p* = 0.657; F = 2.328, *p* = 0.134) and between the groups (F = 2.198, *p* = 0.145; F = 0.777, *p* = 0.383; F = 1.746, *p* = 0.193). Hip abduction strength showed an interaction effect between time and group (F = 4.869, *p* = 0.032) but no significant difference between time points (F = 0.541, *p* = 0.466) and between groups (F = 2.735, *p* = 0.105). Hip adduction strength showed no interaction effect between time and group (F = 1.520, *p* = 0.224) and no significant difference between times (F = 0.113, *p* = 0.738), but there was a significant difference between the groups (F = 4.314, *p* = 0.043). Hip flexion strength showed an interaction effect between time and group (F = 9.218, *p* = 0.004), with a significant difference between times (F = 4.418, *p* = 0.041), but there was no significant difference between the groups (F = 0.060, *p* = 0.807). Hip extension strength showed an interaction effect between time and group (F = 8.386, *p* = 0.006), with a significant difference between times (F = 6.626, *p* = 0.013), but there was no significant difference between the groups (F = 0.167, *p* = 0.085). Hip internal rotation strength showed no interaction between time and group (F = 3.286, *p* = 0.076), and there was no significant difference between the time points (F = 2.558, *p* = 0.117) and between the groups (F = 0.709, *p* = 0.404). Hip external rotation strength showed an interaction (F = 8.578, *p* = 0.005) between the time points and the groups, and there was a significant difference between the time points (F = 7.397, *p* = 0.009), with no significant difference between the groups (F = 2.687, *p* = 0.008). Knee flexion strength showed an interaction effect between time and group (F = 6.220, *p* = 0.016), but there was no significant difference between the time points (F = 0.691, *p* = 0.410) and between the groups (F = 0.128, *p* = 0.722). Knee extension strength showed an effect on the interaction between time and group (F = 5.566, *p* = 0.023), but there was no significant difference between the time points (F = 1.489, *p* = 0.229) and between the groups (F = 0.250, *p* = 0.619).

The sit-up, which evaluates muscular endurance, showed no interaction effect between the time points and the groups (F = 0.067, *p* = 0.798), and there was no significant difference between the times (F = 2.66, *p* = 0.608) and between the groups (F = 0.291, *p* = 0.592).

### 3.3. Changes in Flexibility and Cardiorespiratory Fitness

The results of 10 weeks of online PE classes on flexibility and cardiorespiratory fitness are shown in Table 3. The sit and reach showed no interaction effect between time and group (F = 1.509, *p* = 0.226), and there was no significant difference between the time points (F = 3.264, *p* = 0.077) and between the groups (F = 1.993, *p* = 0.165).

The Harvard step showed no interaction effect between the time points and the groups (F = 2.984, *p* = 0.091), and there was no significant difference between the times (F = 0.220, *p* = 0.641) and between the groups (F= 0.016, *p* = 0.900).

### 3.4. Changes in Balance

The results of the 10 weeks of online PE classes on balance are shown in Table 4. One leg with eyes closed showed no interaction effect between time and group (F = 3.213, *p* = 0.080), and there was no significant difference between the times (F = 1.285, *p* = 0.263) and between the groups (F = 0.000, *p* = 0.990).

The Y-balance test of composite score showed an interaction between time and group (F = 6.497, *p* = 0.014), and there was no significant difference between the time points (F = 0.497, *p* = 0.485). However, there was a significant difference between the groups (F = 6.157, *p* = 0.017).

## 4. Discussion

HIIT is an effective training method to improve physical strength, and several studies have investigated the effects of Tabata training based on high-intensity exercise [19,28,29]. These training methods have been documented as viable for adolescents and adults [20,30]. The situation of COVID-19 requires a survey on the effects of transitions in the PE class environment of Korean middle school students, from face-to-face to online classes. Therefore, the purpose of this study was to investigate the effect of synchronous online PE classes for Tabata training and PE-centered asynchronous online class on the physical strength of adolescents. For the first time, we designed a new study based on Tabata training adopted by Domaradzki et al. [20] and established the relationship between the effects of Tabata training exercise in online classes and the physical fitness of adolescents. The present study showed that the synchronous online class, which was conducted on Tabata training twice a week for 10 weeks, had positive effects on body composition, muscle strength, and balance.

Tabata training based on HIIT was effective in changing the body composition of adolescents, and it was more effective in changing the body composition than obesity prevention programs and physical activity interventions [31]. Racil et al. [32] reported that the 10-week HIIT was more effective than moderate-intensity interval training in the body composition of obese adolescents. Domaradzki et al. [20] reported that high-intensity Tabata training for 10 weeks had a positive effect on the body composition of overweight adolescents and body composition changes in normal-weight adolescents. This study was conducted to verify the effect of high-intensity Tabata training, which is centered on resistance exercise, on adolescents who have normal weight in online PE classes. As a result of checking the body composition after 10 weeks of online PE classes, the interaction effect between the times and the groups was shown in the muscle mass, and a significant difference between the time points, BMI, and body fat was confirmed. Therefore, Tabata training positively affects body composition in online PE classes, a finding that is similar to previous studies. However, unlike previous studies, the BMI and body fat in both groups increased; the increase is believed to be related to a decrease in overall PA and changes in the eating habits of adolescents [16]. In particular, the reason for the increase in BMI, body fat, and muscle mass in SOCG is the Tabata exercise program since there was no difference in eating habits compared to AOCG; however, there is a possibility that the muscle mass increased after exercise. Based on our findings, it is necessary to specifically review the variables that have the greatest influence on body composition during COVID-19 in future studies by adding PA and eating habits.

Lee et al. [6] reported that the hip and knee strength of Korean adolescents decreased during the COVID-19 pandemic. A decrease in muscle strength in adolescence has been shown to affect the increase in muscle-to-fat mass ratio (MFR) [33], deformation of the musculoskeletal system [34], and cardiovascular disease and mortality in middle age [35]. Therefore, online PE classes should be conducted to maintain the physical activity of adolescents, even during COVID-19. Our study focused on improving the physical fitness of adolescents, especially their strength. It focused on increasing vigorous PA, which is pointed out as a disadvantage in online classes. The results confirmed that there was a difference in muscle strength according to the teaching method used. Particularly, the hip and knee muscle strength improved in the SOCG. In previous studies, Tabata training was applied to school PE classes, and it was effective in improving the strength and physical fitness of the students participating in the study [20,30]. However, this study applied Tabata training to online PE classes for the first time. This proved that Tabata training effectively maintains and improves students’ muscular strength in online PE classes. Moliner-Urdiales [36] emphasized high-intensity PA to strengthen adolescents’ lower body strength and argued that it is necessary to consider PE class time in schools. Our program is suitable for online PE class methods, improving the physical strength of adolescents during COVID-19 as a basis for improving lower body muscle strength. As the research results suggest, out-of-school PA for students should be emphasized, and real-time online physical education classes can be an alternative to increase out-of-school PA.

The Y-balance test requires proper hip and knee strength because it needs the lumbo-pelvic hip complex to be stable during the evaluation and control of the body while maintaining the one-footed stand [37,38]. The balancing ability of adolescents is affected by their physical fitness level [37] and isometric strength of the lower extremities [39], being closely related to lower extremity injuries in adolescents [40]. Particularly, sedentary life can reduce adolescents’ posture control and balance ability [41], and a decrease in physical strength related to balance can weaken the ability to sense body position in space, increasing the duration of compensation exercises for body stabilization. The balance of Korean adolescents decreased during the COVID-19 pandemic period, and there was a difference according to gender [6]. Chtourou et al. [42] should focus on restoring motor function through muscle strength, balance training, and routine activities during COVID-19. Training is needed to recover the damaged balance due to a reduction in exercise and proprioceptive sensation [43,44]. A previous study reported that high-intensity interval-type exercise is effective in improving the dynamic balance ability of the general public [45]; particularly, it is effective in improving the overall reach of YBT in subjects with sedentary type 1 diabetes mellitus [46], and in sedentary university students [47]. We applied the Tabata training based on high-intensity interval training to the online PE class; the YBT test before and after the online class showed no interaction effect between the times and the groups in AN, PM, and PL, but the interaction effect was shown in the composite score.

The goal of PE classes during COVID-19 was to increase the PA of adolescents. Although various online PE methods have been applied to adolescents in Korea, our research results suggest that synchronous online PE classes are more effective in increasing PA and maintaining physical fitness levels in adolescents. When the COVID-19 situation improves slightly, students will return to school and participate in various sports activities. Adequate strength and balance skills are essential to participating in various sports, and our program should be adapted to the youth as a beneficial fitness training to prepare them for post-COVID-19 times to prevent sports injuries.

Our study had potential limitations. First, this study did not investigate PA levels among adolescents. In particular, the fact that there was no effect on cardiorespiratory fitness was related to students’ concentration during in-class participation and the type of exercise program. Second, we did not directly measure the PA level of adolescents, which is also considered a limitation of this study. Third, although various types of online PE classes are being introduced in Korea, this study only analyzed the effects of two online PE methods. Further studies should investigate the amount of change in PA among adolescents during the pandemic, program interventions of various types of exercise on online PE classes, and the effect between genders.

## 5. Conclusions

We applied Tabata training as an online PE teaching method to improve the physical fitness of Korean adolescents during the COVID-19 pandemic. As a result, the physical composition, muscle strength, and balance ability of these adolescents improved. In conclusion, a synchronous online PE class can be considered a suitable online PE method for adolescents during COVID-19. An exercise program of less than 40 min (regular class time is 40 min), a PE teacher’s understanding of exercise, the concentration of students participating in online PE classes, an appropriate network environment and appropriate equipment are required to conduct successful synchronous online classes. Most importantly, the role of physical education teachers in a situation such as COVID-19 must change from that of a physical education instructor to that of a PA planner. In addition, real-time online physical education classes are one way to emphasize out-of-school PA. Further, each country’s policy stance must be consistent to activate out-of-school PA. Further studies should develop an exercise program to be applied to online PE classes other than Tabata training, and a program should be created to check whether the youth participating in online PE classes perform the exercises correctly. Moreover, a future online PE system should be established to prepare for the upcoming second wave of COVID-19.

## Figures and Tables

**Table 1:** Changes in body composition.

Variables	Groups	Baseline	Post	F		*p*	*ES* (ɳ^2^)
BMI (kg/m^2^)	AOCG	22.71 ± 4.01	22.95 ± 3.93	T	4.204	0.046 *	0.005
	SOCG	22.04 ± 2.50	22.19 ± 2.40	G	0.563	0.457	
				T × G	0.240	0.626	
Body fat (%)	AOCG	26.97 ± 9.82	27.66 ± 10.07	T	4.587	0.038 *	0.049
	SOCG	25.99 ± 8.25	26.10 ± 8.10	G	0.234	0.631	
				T × G	2.360	0.131	
Muscle mass (kg)	AOCG	24.56 ± 4.53	24.55 ± 4.45	T	4.732	0.035 *	0.106
	SOCG	23.52 ± 3.68	24.00 ± 3.74	G	0.448	0.507	
				T × G	5.426	0.024 *	

Means ± SD: means and standard deviation; BMI: body mass index; AOCG: asynchronous online class group; SOCG: synchronous online class group; G indicates a main effect of groups and T indicates a time effect; T × G indicates an interaction effect; Asterisks denote statistically significant differences at *p* < 0.05.

**Table 2:** Changes in muscle strength and muscle endurance.

Variables	Groups	Baseline	Post	F		*p*	*ES* (ɳ^2^)
Ankle plantar flexion (N·m/kg)	AOCG	0.16 ± 0.05	0.15 ± 0.05	T	1.192	0.281	0.042
	SOCG	0.18 ± 0.04	0.18 ± 0.04	G	2.198	0.145	
				T × G	2.015	0.163	
Ankle dorsi flexion (N·m/kg)	AOCG	0.21 ± 0.06	0.20 ± 0.06	T	3.795	0.058	0.085
	SOCG	0.22 ± 0.06	0.22 ± 0.05	G	1.206	0.278	
				T × G	4.284	0.044 *	
Ankle Inversion (N·m/kg)	AOCG	0.10 ± 0.04	0.10 ± 0.03	T	0.004	0.951	0.019
	SOCG	0.11 ± 0.03	0.11 ± 0.02	G	0.868	0.351	
				T × G	0.492	0.486	
Ankle eversion (N·m/kg)	AOCG	0.09 ± 0.03	0.08 ± 0.03	T	2.328	0.134	0.048
	SOCG	0.10 ± 0.02	0.10 ± 0.02	G	1.746	0.193	
				T × G	2.328	0.134	
Hip abduction (N·m/kg)	AOCG	0.11 ± 0.01	0.10 ± 0.02	T	0.541	0.466	0.096
	SOCG	0.12 ± 0.03	0.12 ± 0.02	G	2.735	0.105	
				T × G	4.869	0.032 *	
Hip adduction (N·m/kg)	AOCG	0.10 ± 0.02	0.10 ± 0.10	T	0.113	0.738	0.032
	SOCG	0.11 ± 0.02	0.11 ± 0.02	G	4.314	0.043 *	
				T × G	1.520	0.224	
Hip flexion (N·m/kg)	AOCG	0.17 ± 0.04	0.16 ± 0.04	T	4.418	0.041 *	0.167
	SOCG	0.17 ± 0.05	0.17 ± 0.05	G	0.060	0.807	
				T × G	9.218	0.004 **	
Hip extension (N·m/kg)	AOCG	0.21 ± 0.07	0.18 ± 0.06	T	6.626	0.013 *	0.154
	SOCG	0.20 ± 0.05	0.20 ± 0.06	G	0.167	0.685	
				T × G	8.386	0.006 **	
Hip internal rotation (N·m/kg)	AOCG	0.11 ± 0.03	0.11 ± 0.03	T	2.558	0.117	0.067
	SOCG	0.12 ± 0.03	0.12 ± 0.03	G	0.709	0.404	
				T × G	3.286	0.076	
Hip external rotation (N·m/kg)	AOCG	0.11 ± 0.03	0.10 ± 0.02	T	7.397	0.009 **	0.157
	SOCG	0.11 ± 0.01	0.11 ± 0.02	G	2.687	0.108	
				T × G	8.578	0.005 **	
Knee flexion (N·m/kg)	AOCG	0.20 ± 0.05	0.19 ± 0.05	T	0.691	0.410	0.119
	SOCG	0.20 ± 0.06	0.21 ± 0.07	G	0.128	0.722	
				T × G	6.220	0.016 *	
Knee extension (N·m/kg)	AOCG	0.24 ± 0.10	0.23 ± 0.09	T	1.489	0.229	0.108
	SOCG	0.25 ± 0.07	0.25 ± 0.07	G	0.250	0.619	
				T × G	5.566	0.023 *	
Sit-up (rep.)	AOCG	15.54 ± 5.01	15.41 ± 4.73	T	2.455	0.124	0.076
	SOCG	16.29 ± 5.51	17.45 ± 5.86	G	0.874	0.355	
				T × G	3.775	0.058	

Values are means ± SD: means and standard deviation; AOCG: asynchronous online class group; SOCG: synchronous online class group; G indicates a main effect of groups and T indicates a time effect; T × G indicates an interaction effect; Asterisks denote statistically significant differences at * *p* < 0.05 and ** *p* < 0.01.

**Table 3:** Changes in flexibility and cardiorespiratory fitness.

Variables	Groups	Baseline	Post	F		*p*	*ES* (ɳ^2^)
Sit and reach (cm)	AOCG	7.28 ± 9.65	6.76 ± 9.31	T	3.264	0.077	0.032
	SOCG	10.66 ± 8.18	10.56 ± 8.02	G	1.993	0.165	
				T × G	1.509	0.226	
Harvard step (%)	AOCG	46.19 ± 9.43	45.55 ± 7.72	T	0.220	0.641	0.061
	SOCG	45.64 ± 9.94	46.75 ± 9.11	G	0.016	0.900	
				T × G	2.984	0.091	

Values are means ± SD: means and standard deviation; AOCG: asynchronous online class group; SOCG: synchronous online class group; G indicates a main effect of groups, and T indicates a time effect; T × G indicates an interaction effect.

**Table 4:** Changes in balance.

Variables	Groups	Baseline	Post	F		*p*	*ES* (ɳ^2^)
One leg with eyes close (sec)	AOCG	11.12 ± 6.34	10.87 ± 5.45	T	1.285	0.263	0.065
	SOCG	10.48 ± 5.44	11.55 ± 5.20	G	0.000	0.990	
				T × G	3.213	0.080	
Y-Balance test composite (%)	AOCG	87.42 ± 10.51	85.23 ± 9.14	T	0.497	0.485	0.124
	SOCG	92.50 ± 9.65	93.74 ± 9.69	G	6.157	0.017 *	
				T × G	6.497	0.014 *	

Values are means ± SD: means and standard deviation; AOCG: asynchronous online class group; SOCG: synchronous online class group; G indicates a main effect of groups and T indicates a time effect; T × G indicates an interaction effect; Asterisks denote statistically significant differences at *p* < 0.05.

## Data Availability

The data presented in this study are available upon request from the corresponding author.

## Supplementary Materials

The following are available online at https://www.mdpi.com/article/10.3390/ijerph181910305/s1, Figure S1: Consolidated Standards of Reporting Trials flow diagram, Table S1: Exercise protocols.
