# Supplementary material for: Impact of Synchronous Online Physical Education Classes Using Tabata Training on Adolescents during COVID-19: A Randomized Controlled Study
Source: Int J Environ Res Public Health. 2021 Sep 30;18(19):10305. doi: 10.3390/ijerph181910305 (PMC8507984; doi:10.3390/ijerph181910305)
Supplement: Supplementary file 1 [file ijerph-18-10305-s001.zip › Table S1_R1.pdf]

Table S1. Exercise protocols

1. Asynchronous online class (content-focused class)

	subject	contents
1 weeks	Basic skills and concepts of volleyball	- Introduction to volleyball (history, characteristic, rule)
2 weeks		- Introduction to basic volleyball terms and skills<br>(pass, set, dig, serve, spike)
3 weeks		- Learnnig skills of attacking, blocking, and defending skill<br>- Watching video (volleyball game)
4 weeks	Basic skills and concepts of basketball	- Introduction to volleyball (history, characteristic, rule)
5 weeks		- Introduction to basic basketball terms and skills<br>(pass, dribble, Lay-up, shot)
6 weeks		- Learnnig skills of attacking and defending skill<br>- Watching video (basketball game)
7 weeks	Heath management	- Healthy lifestyle education<br>- Healthy dietary education<br>- Understanding physical fitness<br>(health-related physical fitness and skill-related physical fitness)<br>- Tabata training
8 weeks		
9 weeks	school safety education	- Water-related safety education<br>- First-Aid education<br>- Safety accidents in physical education class
10 weeks		

2. Synchronous online class (real-time bilateral class)

Weeks 1~10

	Exercise		Position	Reps or time	Rest	Load	RPE
Warm-up (10 min)	Dynamic stretching		Standing	10 min	-	-	6~7
Tabata training (14 min)	Low exercise intensity	Narrow stance squat	Standing	20 sec	1 min	BW	1~2 weeks: 12~14,<br><br>3~10 weeks : 15~
		Butt kicker	Standing				
		Toe touches	Supine				
		Lunges	Standing				
		Mountain climbers	prone				
		Jumping jacks	Standing				
		Standing abs twist	Standing				
		Squat to side	Standing				
	Maximum exercise intensity	Walking	Standing	10 sec			8~10
Cool-down (10 min)	Static		Standing & Sitting	10 min	-	-	6

BW: body weight, RPE: rating of perceived exertion
