# Supplementary figures and images for: Impact of Synchronous Online Physical Education Classes Using Tabata Training on Adolescents during COVID-19: A Randomized Controlled Study
Source: Int J Environ Res Public Health. 2021 Sep 30;18(19):10305. doi: 10.3390/ijerph181910305 (PMC8507984; doi:10.3390/ijerph181910305)

## CONSORT 2020 Flow Diagram

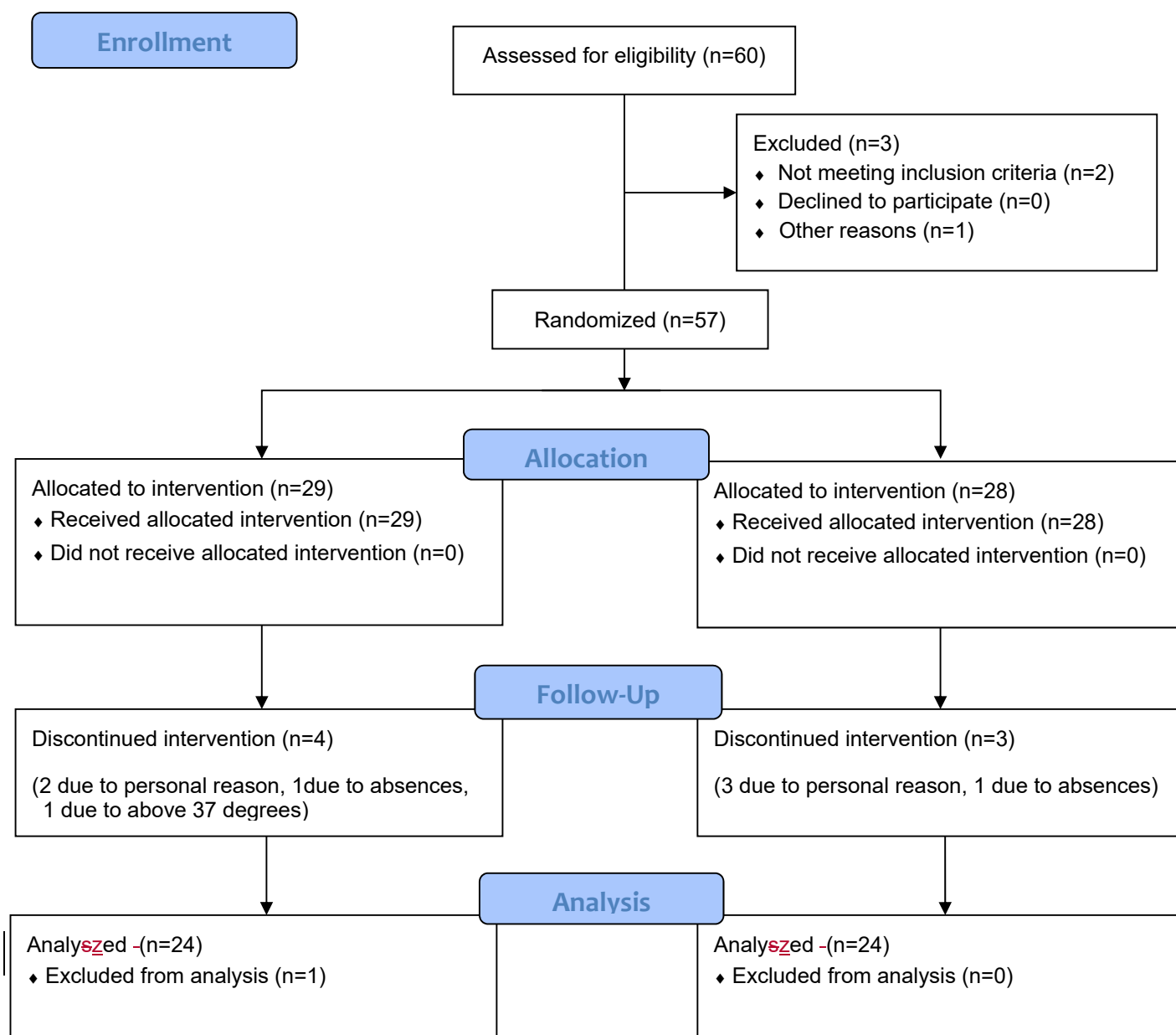

Supplement: Supplementary file 1 [file ijerph-18-10305-s001.zip › Figure S1.pdf]
